# Supplementary figures and images for: Variability within a clonal population of Erwinia amylovora disclosed by phenotypic analysis
Source: PeerJ. 2022 Jul 21;10:e13695. doi: 10.7717/peerj.13695 (PMC9308965; doi:10.7717/peerj.13695)

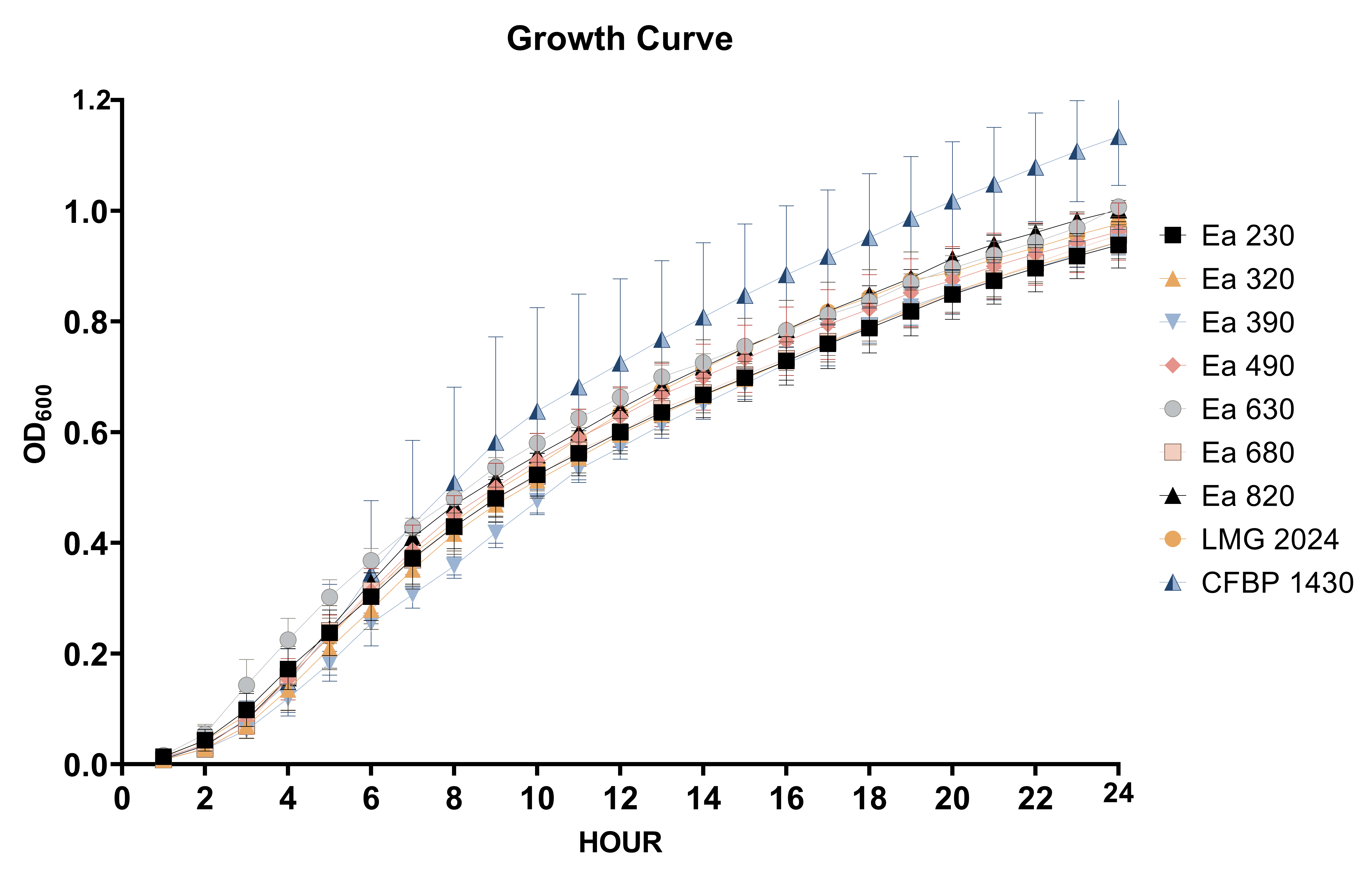

Supplement: Supplemental Information 1 [file peerj-10-13695-s001.png]
