# Supplementary material for: Variability within a clonal population of Erwinia amylovora disclosed by phenotypic analysis
Source: PeerJ. 2022 Jul 21;10:e13695. doi: 10.7717/peerj.13695 (PMC9308965; doi:10.7717/peerj.13695)
Supplement: Supplemental Information 2 [file peerj-10-13695-s002.docx]

**Table S1.** Bacterial strains retrieved from NCBI used for MLSA analysis.

| Strain | Origin | Host | Isolation Year | Accession number |
| --- | --- | --- | --- | --- |
| *Erwinia amylovora* FB-20 | South Korea | Pear | 2015 | [CP050240](https://www.ncbi.nlm.nih.gov/nucleotide/CP050240.1?report=genbank&log$=nucltop&blast_rank=1&RID=HGA8NVMV014) |
| *Erwinia amylovora* FB-86 | South Korea | Apple | 2015 | [CP050258](https://www.ncbi.nlm.nih.gov/nucleotide/CP050258.1?report=genbank&log$=nucltop&blast_rank=2&RID=HGA8NVMV014) |
| *Erwinia amylovora* FB-207 | South Korea | Pear | 2015 | [CP050263](https://www.ncbi.nlm.nih.gov/nucleotide/CP050263.1?report=genbank&log$=nucltop&blast_rank=3&RID=HGA8NVMV014) |
| *Erwinia amylovora* FB-307 | South Korea | Apple | 2015 | [CP050242](https://www.ncbi.nlm.nih.gov/nucleotide/CP050242.1?report=genbank&log$=nucltop&blast_rank=4&RID=HGA8NVMV014) |
| *Erwinia amylovora* TS3238 | South Korea | Pear | 2015 | [CP050244](https://www.ncbi.nlm.nih.gov/nucleotide/CP050244.1?report=genbank&log$=nucltop&blast_rank=5&RID=HGA8NVMV014) |
| *Erwinia amylovora* E-2 | Belarus | Apple | 2007 | [CP024970](https://www.ncbi.nlm.nih.gov/nucleotide/CP024970.1?report=genbank&log$=nucltop&blast_rank=7&RID=HGA8NVMV014) |
| *Erwinia amylovora* TS3128 | South Korea | Pear | 2015 | [CP056034](https://www.ncbi.nlm.nih.gov/nucleotide/CP056034.1?report=genbank&log$=nucltop&blast_rank=8&RID=HGA8NVMV014) |
| *Erwinia amylovora* CFBP1430 | France | *Crataegus oxyacantha* | 1972 | [FN434113](https://www.ncbi.nlm.nih.gov/nucleotide/FN434113.1?report=genbank&log$=nucltop&blast_rank=10&RID=HGA8NVMV014) |
| *Erwinia amylovora* ATCC 49946 | USA | Apple | Unknown | [FN666575](https://www.ncbi.nlm.nih.gov/nucleotide/FN666575.1?report=genbank&log$=nucltop&blast_rank=11&RID=HGA8NVMV014) |
| *Erwinia amylovora* CTBT1-1 | USA | Pear | 2015 | NQJP01000001 |
| *Erwinia amylovora* CTBT3-1 | USA | Pear | 2015 | NQJO01000001 |
| *Erwinia amylovora* Ea1/79Sm | Germany | Apple | 1979 | CP064855 |
| *Erwinia amylovora* Ea1189 | USA | Apple | 2018 | CP055227 |
| *Erwinia amylovora* MAGFLF 2 | USA | Apple | 2015 | NQJN01000001 |
| *Erwinia amylovora* RISTBO01-2 | USA | Apple | 2015 | NQJM01000001 |
| *Erwinia amylovora* VTDMSF02 | USA | Apple | 2015 | NQJL01000001 |
| *Erwinia amylovora* LMG 2024 | United Kingdom | Pear | 1959 | NZ_CAPB01000000 |
| *Erwinia pyrifoliae DSM 12163* | South Korea | Unknown | <1998 | NC_017390 |
| *Erwinia rhapontici BIGb0435* | United Kingdom | *Rheum rhaponticum* | <1988 | NZ_SOAQ00000000 |
| *Erwinia tasmaniensis ET1/99* | Australia | Apple | 1999 | NC_010694 |
| *Erwinia billingiae Eb661* | United Kingdom | Pear | 1959 | NC_014306 |
