# Supplementary material for: Variability within a clonal population of Erwinia amylovora disclosed by phenotypic analysis
Source: PeerJ. 2022 Jul 21;10:e13695. doi: 10.7717/peerj.13695 (PMC9308965; doi:10.7717/peerj.13695)
Supplement: Supplemental Information 3 [file peerj-10-13695-s003.docx]

**Table S2.** List of Portuguese Erwinia amylovora strains used and the correspondent GenBank accession number of gyrB, gapA, rpoD and infB regions.

| Strain | *gapA* Accession Number | *gyrB* Accession Number | *infB* Accession Number | *rpoD* Accession Number |
| --- | --- | --- | --- | --- |
| Ea 230 | MW647223 | MW647230 | MW647237 | MW647244 |
| Ea 320 | MW647224 | MW647231 | MW647238 | MW647245 |
| Ea 390 | MW647225 | MW647232 | MW647239 | MW647246 |
| Ea 490 | MW647226 | MW647233 | MW647240 | MW647247 |
| Ea 630 | MW647227 | MW647234 | MW647241 | MW647248 |
| Ea 680 | MW647228 | MW647235 | MW647242 | MW647249 |
| Ea 820 | MW647229 | MW647236 | MW647243 | MW647250 |
