# Supplementary material for: Variability within a clonal population of Erwinia amylovora disclosed by phenotypic analysis
Source: PeerJ. 2022 Jul 21;10:e13695. doi: 10.7717/peerj.13695 (PMC9308965; doi:10.7717/peerj.13695)
Supplement: Supplemental Information 4 — Every sequence is presented 5′–3′ [file peerj-10-13695-s004.docx]

**Table S3.** Sequences of gapA, gyrB, infB and rpoD of the Portuguese Erwinia amylovora strains used in this work. Every sequence is presented 5´- 3´.

| **Strain** | **Ea 230** | **Ea 320** | **Ea 390** | **Ea 490** | **Ea 630** | **Ea 680** | **Ea 820** |
| --- | --- | --- | --- | --- | --- | --- | --- |
| ***gapA* Sequence** | TCGACGCAGATTACATGGCTTACATGCTGAAGTATGACTCAACTCATGGTCGCTTTAACGGCACTGTAGAAGTCAAAGACGGCCATTTGGTTGTTAACGGCAAAACCATCCGCGTTACTGCTGAGCGCGATCCGGCGAACCTGAAGTGGGATGCGGTTGGCGTTGATGTGGTTGCTGAAGCAACCGGTATCTTCCTGACCGATGAAACTGCTCGTAAACACATCGAAGCTGGCGCTAAGAAAGTGGTACTGACTGGTCCGTCCAAGGATGATACCCCGATGTTCGTAATGGGGGTTAACCACAAGGCTTACGCGGGTCAGGCTATCGTTTCTAACGCATCCTGCACCACCAACTGCCTGGCACCGCTGGCGAAAGTGATCAACGATAAATTCGGTATCGTTGAAGCATTGATGACCACGGTACACGCCACTACGGCGACTCAGAAAACCGTTGACGGCCCGTCTCACAAAGACTGGCGCGGCGGCCGTGGCGCATCTCAGAACATTATCCCTTCTTCTACCGGTGCGGCTAAAGCGGTTGGAAAAGTGATCCCTGAGTT | TCGACGCAGATTACATGGCTTACATGCTGAAGTATGACTCAACTCATGGTCGCTTTAACGGCACTGTAGAAGTCAAAGACGGCCATTTGGTTGTTAACGGCAAAACCATCCGCGTTACTGCTGAGCGCGATCCGGCGAACCTGAAGTGGGATGCGGTTGGCGTTGATGTGGTTGCTGAAGCAACCGGTATCTTCCTGACCGATGAAACTGCTCGTAAACACATCGAAGCTGGCGCTAAGAAAGTGGTACTGACTGGTCCGTCCAAGGATGATACCCCGATGTTCGTAATGGGGGTTAACCACAAGGCTTACGCGGGTCAGGCTATCGTTTCTAACGCATCCTGCACCACCAACTGCCTGGCACCGCTGGCGAAAGTGATCAACGATAAATTCGGTATCGTTGAAGCATTGATGACCACGGTACACGCCACTACGGCGACTCAGAAAACCGTTGACGGCCCGTCTCACAAAGACTGGCGCGGCGGCCGTGGCGCATCTCAGAACATTATCCCTTCTTCTACCGGTGCGGCTAAAGCGGTTGGAAAAGTGATCCCTGAGTT | TCGACGCAGATTACATGGCTTACATGCTGAAGTATGACTCAACTCATGGTCGCTTTAACGGCACTGTAGAAGTCAAAGACGGCCATTTGGTTGTTAACGGCAAAACCATCCGCGTTACTGCTGAGCGCGATCCGGCGAACCTGAAGTGGGATGCGGTTGGCGTTGATGTGGTTGCTGAAGCAACCGGTATCTTCCTGACCGATGAAACTGCTCGTAAACACATCGAAGCTGGCGCTAAGAAAGTGGTACTGACTGGTCCGTCCAAGGATGATACCCCGATGTTCGTAATGGGGGTTAACCACAAGGCTTACGCGGGTCAGGCTATCGTTTCTAACGCATCCTGCACCACCAACTGCCTGGCACCGCTGGCGAAAGTGATCAACGATAAATTCGGTATCGTTGAAGCATTGATGACCACGGTACACGCCACTACGGCGACTCAGAAAACCGTTGACGGCCCGTCTCACAAAGACTGGCGCGGCGGCCGTGGCGCATCTCAGAACATTATCCCTTCTTCTACCGGTGCGGCTAAAGCGGTTGGAAAAGTGATCCCTGAGTT | TCGACGCAGATTACATGGCTTACATGCTGAAGTATGACTCAACTCATGGTCGCTTTAACGGCACTGTAGAAGTCAAAGACGGCCATTTGGTTGTTAACGGCAAAACCATCCGCGTTACTGCTGAGCGCGATCCGGCGAACCTGAAGTGGGATGCGGTTGGCGTTGATGTGGTTGCTGAAGCAACCGGTATCTTCCTGACCGATGAAACTGCTCGTAAACACATCGAAGCTGGCGCTAAGAAAGTGGTACTGACTGGTCCGTCCAAGGATGATACCCCGATGTTCGTAATGGGGGTTAACCACAAGGCTTACGCGGGTCAGGCTATCGTTTCTAACGCATCCTGCACCACCAACTGCCTGGCACCGCTGGCGAAAGTGATCAACGATAAATTCGGTATCGTTGAAGCATTGATGACCACGGTACACGCCACTACGGCGACTCAGAAAACCGTTGACGGCCCGTCTCACAAAGACTGGCGCGGCGGCCGTGGCGCATCTCAGAACATTATCCCTTCTTCTACCGGTGCGGCTAAAGCGGTTGGAAAAGTGATCCCTGAGTT | TCGACGCAGATTACATGGCTTACATGCTGAAGTATGACTCAACTCATGGTCGCTTTAACGGCACTGTAGAAGTCAAAGACGGCCATTTGGTTGTTAACGGCAAAACCATCCGCGTTACTGCTGAGCGCGATCCGGCGAACCTGAAGTGGGATGCGGTTGGCGTTGATGTGGTTGCTGAAGCAACCGGTATCTTCCTGACCGATGAAACTGCTCGTAAACACATCGAAGCTGGCGCTAAGAAAGTGGTACTGACTGGTCCGTCCAAGGATGATACCCCGATGTTCGTAATGGGGGTTAACCACAAGGCTTACGCGGGTCAGGCTATCGTTTCTAACGCATCCTGCACCACCAACTGCCTGGCACCGCTGGCGAAAGTGATCAACGATAAATTCGGTATCGTTGAAGCATTGATGACCACGGTACACGCCACTACGGCGACTCAGAAAACCGTTGACGGCCCGTCTCACAAAGACTGGCGCGGCGGCCGTGGCGCATCTCAGAACATTATCCCTTCTTCTACCGGTGCGGCTAAAGCGGTTGGAAAAGTGATCCCTGAGTT | TCGACGCAGATTACATGGCTTACATGCTGAAGTATGACTCAACTCATGGTCGCTTTAACGGCACTGTAGAAGTCAAAGACGGCCATTTGGTTGTTAACGGCAAAACCATCCGCGTTACTGCTGAGCGCGATCCGGCGAACCTGAAGTGGGATGCGGTTGGCGTTGATGTGGTTGCTGAAGCAACCGGTATCTTCCTGACCGATGAAACTGCTCGTAAACACATCGAAGCTGGCGCTAAGAAAGTGGTACTGACTGGTCCGTCCAAGGATGATACCCCGATGTTCGTAATGGGGGTTAACCACAAGGCTTACGCGGGTCAGGCTATCGTTTCTAACGCATCCTGCACCACCAACTGCCTGGCACCGCTGGCGAAAGTGATCAACGATAAATTCGGTATCGTTGAAGCATTGATGACCACGGTACACGCCACTACGGCGACTCAGAAAACCGTTGACGGCCCGTCTCACAAAGACTGGCGCGGCGGCCGTGGCGCATCTCAGAACATTATCCCTTCTTCTACCGGTGCGGCTAAAGCGGTTGGAAAAGTGATCCCTGAGTT | TCGACGCAGATTACATGGCTTACATGCTGAAGTATGACTCAACTCATGGTCGCTTTAACGGCACTGTAGAAGTCAAAGACGGCCATTTGGTTGTTAACGGCAAAACCATCCGCGTTACTGCTGAGCGCGATCCGGCGAACCTGAAGTGGGATGCGGTTGGCGTTGATGTGGTTGCTGAAGCAACCGGTATCTTCCTGACCGATGAAACTGCTCGTAAACACATCGAAGCTGGCGCTAAGAAAGTGGTACTGACTGGTCCGTCCAAGGATGATACCCCGATGTTCGTAATGGGGGTTAACCACAAGGCTTACGCGGGTCAGGCTATCGTTTCTAACGCATCCTGCACCACCAACTGCCTGGCACCGCTGGCGAAAGTGATCAACGATAAATTCGGTATCGTTGAAGCATTGATGACCACGGTACACGCCACTACGGCGACTCAGAAAACCGTTGACGGCCCGTCTCACAAAGACTGGCGCGGCGGCCGTGGCGCATCTCAGAACATTATCCCTTCTTCTACCGGTGCGGCTAAAGCGGTTGGAAAAGTGATCCCTGAGTT |
| ***gyrB* Sequence** | ACGGCGTGGGTGTTTCCGTGGTTAACGCCCTGTCGGAAAAACTGGAACTGACCATCCGCCGTGAAGGCAAAGTCCACCAGCAGGTCTACGTTCACGGCGTGCCACAGGCCGCGCTGAATGTCACCGGCGAAACCGATTTGACCGGAACGCGGGTGCGTTTCTGGCCTAGTCATCAAACCTTTACTAACGTTGTTGAATTCGAATACGAAATACTGGCAAAACGTCTGCGTGAGCTGTCGTTCCTGAACTCCGGCGTATCCATCAAGCTGGAAGATAAGCGTGACGGCAAAAACGACCATTACCACTACGAAGGTGGTATCAAAGCGTTCGTTGAGTACCTGAACAAAAACAAAACCCCGATCCACCCCAACGTGTTCTATTTCTCCACCGAGAAAGATGGCATCGGCGTGGAAGTGGCCCTGCAGTGGAACGATGGTTTCCAGGAAAATATCTACTGCTTTACCAACAATATTCCGCAGCGCGACGGCGGTACGCACCTGGCGGGCTTCCGTGCCGCCATGACCCGCACCCTGAACGCCTACATG | ACGGCGTGGGTGTTTCCGTGGTTAACGCCCTGTCGGAAAAACTGGAACTGACCATCCGCCGTGAAGGCAAAGTCCACCAGCAGGTCTACGTTCACGGCGTGCCACAGGCCGCGCTGAATGTCACCGGCGAAACCGATTTGACCGGAACGCGGGTGCGTTTCTGGCCTAGTCATCAAACCTTTACTAACGTTGTTGAATTCGAATACGAAATACTGGCAAAACGTCTGCGTGAGCTGTCGTTCCTGAACTCCGGCGTATCCATCAAGCTGGAAGATAAGCGTGACGGCAAAAACGACCATTACCACTACGAAGGTGGTATCAAAGCGTTCGTTGAGTACCTGAACAAAAACAAAACCCCGATCCACCCCAACGTGTTCTATTTCTCCACCGAGAAAGATGGCATCGGCGTGGAAGTGGCCCTGCAGTGGAACGATGGTTTCCAGGAAAATATCTACTGCTTTACCAACAATATTCCGCAGCGCGACGGCGGTACGCACCTGGCGGGCTTCCGTGCCGCCATGACCCGCACCCTGAACGCCTACATG | ACGGCGTGGGTGTTTCCGTGGTTAACGCCCTGTCGGAAAAACTGGAACTGACCATCCGCCGTGAAGGCAAAGTCCACCAGCAGGTCTACGTTCACGGCGTGCCACAGGCCGCGCTGAATGTCACCGGCGAAACCGATTTGACCGGAACGCGGGTGCGTTTCTGGCCTAGTCATCAAACCTTTACTAACGTTGTTGAATTCGAATACGAAATACTGGCAAAACGTCTGCGTGAGCTGTCGTTCCTGAACTCCGGCGTATCCATCAAGCTGGAAGATAAGCGTGACGGCAAAAACGACCATTACCACTACGAAGGTGGTATCAAAGCGTTCGTTGAGTACCTGAACAAAAACAAAACCCCGATCCACCCCAACGTGTTCTATTTCTCCACCGAGAAAGATGGCATCGGCGTGGAAGTGGCCCTGCAGTGGAACGATGGTTTCCAGGAAAATATCTACTGCTTTACCAACAATATTCCGCAGCGCGACGGCGGTACGCACCTGGCGGGCTTCCGTGCCGCCATGACCCGCACCCTGAACGCCTACATG | ACGGCGTGGGTGTTTCCGTGGTTAACGCCCTGTCGGAAAAACTGGAACTGACCATCCGCCGTGAAGGCAAAGTCCACCAGCAGGTCTACGTTCACGGCGTGCCACAGGCCGCGCTGAATGTCACCGGCGAAACCGATTTGACCGGAACGCGGGTGCGTTTCTGGCCTAGTCATCAAACCTTTACTAACGTTGTTGAATTCGAATACGAAATACTGGCAAAACGTCTGCGTGAGCTGTCGTTCCTGAACTCCGGCGTATCCATCAAGCTGGAAGATAAGCGTGACGGCAAAAACGACCATTACCACTACGAAGGTGGTATCAAAGCGTTCGTTGAGTACCTGAACAAAAACAAAACCCCGATCCACCCCAACGTGTTCTATTTCTCCACCGAGAAAGATGGCATCGGCGTGGAAGTGGCCCTGCAGTGGAACGATGGTTTCCAGGAAAATATCTACTGCTTTACCAACAATATTCCGCAGCGCGACGGCGGTACGCACCTGGCGGGCTTCCGTGCCGCCATGACCCGCACCCTGAACGCCTACATG | ACGGCGTGGGTGTTTCCGTGGTTAACGCCCTGTCGGAAAAACTGGAACTGACCATCCGCCGTGAAGGCAAAGTCCACCAGCAGGTCTACGTTCACGGCGTGCCACAGGCCGCGCTGAATGTCACCGGCGAAACCGATTTGACCGGAACGCGGGTGCGTTTCTGGCCTAGTCATCAAACCTTTACTAACGTTGTTGAATTCGAATACGAAATACTGGCAAAACGTCTGCGTGAGCTGTCGTTCCTGAACTCCGGCGTATCCATCAAGCTGGAAGATAAGCGTGACGGCAAAAACGACCATTACCACTACGAAGGTGGTATCAAAGCGTTCGTTGAGTACCTGAACAAAAACAAAACCCCGATCCACCCCAACGTGTTCTATTTCTCCACCGAGAAAGATGGCATCGGCGTGGAAGTGGCCCTGCAGTGGAACGATGGTTTCCAGGAAAATATCTACTGCTTTACCAACAATATTCCGCAGCGCGACGGCGGTACGCACCTGGCGGGCTTCCGTGCCGCCATGACCCGCACCCTGAACGCCTACATG | ACGGCGTGGGTGTTTCCGTGGTTAACGCCCTGTCGGAAAAACTGGAACTGACCATCCGCCGTGAAGGCAAAGTCCACCAGCAGGTCTACGTTCACGGCGTGCCACAGGCCGCGCTGAATGTCACCGGCGAAACCGATTTGACCGGAACGCGGGTGCGTTTCTGGCCTAGTCATCAAACCTTTACTAACGTTGTTGAATTCGAATACGAAATACTGGCAAAACGTCTGCGTGAGCTGTCGTTCCTGAACTCCGGCGTATCCATCAAGCTGGAAGATAAGCGTGACGGCAAAAACGACCATTACCACTACGAAGGTGGTATCAAAGCGTTCGTTGAGTACCTGAACAAAAACAAAACCCCGATCCACCCCAACGTGTTCTATTTCTCCACCGAGAAAGATGGCATCGGCGTGGAAGTGGCCCTGCAGTGGAACGATGGTTTCCAGGAAAATATCTACTGCTTTACCAACAATATTCCGCAGCGCGACGGCGGTACGCACCTGGCGGGCTTCCGTGCCGCCATGACCCGCACCCTGAACGCCTACATG | ACGGCGTGGGTGTTTCCGTGGTTAACGCCCTGTCGGAAAAACTGGAACTGACCATCCGCCGTGAAGGCAAAGTCCACCAGCAGGTCTACGTTCACGGCGTGCCACAGGCCGCGCTGAATGTCACCGGCGAAACCGATTTGACCGGAACGCGGGTGCGTTTCTGGCCTAGTCATCAAACCTTTACTAACGTTGTTGAATTCGAATACGAAATACTGGCAAAACGTCTGCGTGAGCTGTCGTTCCTGAACTCCGGCGTATCCATCAAGCTGGAAGATAAGCGTGACGGCAAAAACGACCATTACCACTACGAAGGTGGTATCAAAGCGTTCGTTGAGTACCTGAACAAAAACAAAACCCCGATCCACCCCAACGTGTTCTATTTCTCCACCGAGAAAGATGGCATCGGCGTGGAAGTGGCCCTGCAGTGGAACGATGGTTTCCAGGAAAATATCTACTGCTTTACCAACAATATTCCGCAGCGCGACGGCGGTACGCACCTGGCGGGCTTCCGTGCCGCCATGACCCGCACCCTGAACGCCTACATG |
| ***infB* Sequence** | GAAGCCATCCAGCATGCGAAAGCGGCGAAAGTGCCGGTTGTGGTGGCGGTAAACAAATGTGATAAGCCAGAAGCCGATCCGGACCGTGTCAAAAACGAACTCACCCAGTACGGCATCATTCCGGAAGAGTGGGGCGGTGAAAACATGTTCGTCAACGTCTCTGCGAAAGCCGGTACCGGGATTGATGACTTGCTGAATGCCATCCTGCTGCAGGCGGAAGTTCTTGAACTGACCGCCGTACGTCAGGGCATGGCAAGCGGTGTGGTGATCGAATCCTTCCTGGATAAAGGCCGTGGCCCGGTGGCTACCGTGCTGGTGCGTGAAGGTACGCTGAACAAAGGCGATATCGTGCTGTGTGGTTTCGAATATGGCCGCGTACGAGCAATGCGTGACGAGCTGGGCCGCGAAGTACTGACTGCGGGTCCATCTATCCCGGTTGAGATCCTCGGCATGTCCGGCGTACCGGCGGCGGGTGATGAAGCCACCGTGGTGCGTGACGAGAAGAAAGCGCGTGAAGTGGCGCTGTACCGTCAGGGCAAATTCCGTGAAGTT | GAAGCCATCCAGCATGCGAAAGCGGCGAAAGTGCCGGTTGTGGTGGCGGTAAACAAATGTGATAAGCCAGAAGCCGATCCGGACCGTGTCAAAAACGAACTCACCCAGTACGGCATCATTCCGGAAGAGTGGGGCGGTGAAAACATGTTCGTCAACGTCTCTGCGAAAGCCGGTACCGGGATTGATGACTTGCTGAATGCCATCCTGCTGCAGGCGGAAGTTCTTGAACTGACCGCCGTACGTCAGGGCATGGCAAGCGGTGTGGTGATCGAATCCTTCCTGGATAAAGGCCGTGGCCCGGTGGCTACCGTGCTGGTGCGTGAAGGTACGCTGAACAAAGGCGATATCGTGCTGTGTGGTTTCGAATATGGCCGCGTACGAGCAATGCGTGACGAGCTGGGCCGCGAAGTACTGACTGCGGGTCCATCTATCCCGGTTGAGATCCTCGGCATGTCCGGCGTACCGGCGGCGGGTGATGAAGCCACCGTGGTGCGTGACGAGAAGAAAGCGCGTGAAGTGGCGCTGTACCGTCAGGGCAAATTCCGTGAAGTT | GAAGCCATCCAGCATGCGAAAGCGGCGAAAGTGCCGGTTGTGGTGGCGGTAAACAAATGTGATAAGCCAGAAGCCGATCCGGACCGTGTCAAAAACGAACTCACCCAGTACGGCATCATTCCGGAAGAGTGGGGCGGTGAAAACATGTTCGTCAACGTCTCTGCGAAAGCCGGTACCGGGATTGATGACTTGCTGAATGCCATCCTGCTGCAGGCGGAAGTTCTTGAACTGACCGCCGTACGTCAGGGCATGGCAAGCGGTGTGGTGATCGAATCCTTCCTGGATAAAGGCCGTGGCCCGGTGGCTACCGTGCTGGTGCGTGAAGGTACGCTGAACAAAGGCGATATCGTGCTGTGTGGTTTCGAATATGGCCGCGTACGAGCAATGCGTGACGAGCTGGGCCGCGAAGTACTGACTGCGGGTCCATCTATCCCGGTTGAGATCCTCGGCATGTCCGGCGTACCGGCGGCGGGTGATGAAGCCACCGTGGTGCGTGACGAGAAGAAAGCGCGTGAAGTGGCGCTGTACCGTCAGGGCAAATTCCGTGAAGTT | GAAGCCATCCAGCATGCGAAAGCGGCGAAAGTGCCGGTTGTGGTGGCGGTAAACAAATGTGATAAGCCAGAAGCCGATCCGGACCGTGTCAAAAACGAACTCACCCAGTACGGCATCATTCCGGAAGAGTGGGGCGGTGAAAACATGTTCGTCAACGTCTCTGCGAAAGCCGGTACCGGGATTGATGACTTGCTGAATGCCATCCTGCTGCAGGCGGAAGTTCTTGAACTGACCGCCGTACGTCAGGGCATGGCAAGCGGTGTGGTGATCGAATCCTTCCTGGATAAAGGCCGTGGCCCGGTGGCTACCGTGCTGGTGCGTGAAGGTACGCTGAACAAAGGCGATATCGTGCTGTGTGGTTTCGAATATGGCCGCGTACGAGCAATGCGTGACGAGCTGGGCCGCGAAGTACTGACTGCGGGTCCATCTATCCCGGTTGAGATCCTCGGCATGTCCGGCGTACCGGCGGCGGGTGATGAAGCCACCGTGGTGCGTGACGAGAAGAAAGCGCGTGAAGTGGCGCTGTACCGTCAGGGCAAATTCCGTGAAGTT | GAAGCCATCCAGCATGCGAAAGCGGCGAAAGTGCCGGTTGTGGTGGCGGTAAACAAATGTGATAAGCCAGAAGCCGATCCGGACCGTGTCAAAAACGAACTCACCCAGTACGGCATCATTCCGGAAGAGTGGGGCGGTGAAAACATGTTCGTCAACGTCTCTGCGAAAGCCGGTACCGGGATTGATGACTTGCTGAATGCCATCCTGCTGCAGGCGGAAGTTCTTGAACTGACCGCCGTACGTCAGGGCATGGCAAGCGGTGTGGTGATCGAATCCTTCCTGGATAAAGGCCGTGGCCCGGTGGCTACCGTGCTGGTGCGTGAAGGTACGCTGAACAAAGGCGATATCGTGCTGTGTGGTTTCGAATATGGCCGCGTACGAGCAATGCGTGACGAGCTGGGCCGCGAAGTACTGACTGCGGGTCCATCTATCCCGGTTGAGATCCTCGGCATGTCCGGCGTACCGGCGGCGGGTGATGAAGCCACCGTGGTGCGTGACGAGAAGAAAGCGCGTGAAGTGGCGCTGTACCGTCAGGGCAAATTCCGTGAAGTT | GAAGCCATCCAGCATGCGAAAGCGGCGAAAGTGCCGGTTGTGGTGGCGGTAAACAAATGTGATAAGCCAGAAGCCGATCCGGACCGTGTCAAAAACGAACTCACCCAGTACGGCATCATTCCGGAAGAGTGGGGCGGTGAAAACATGTTCGTCAACGTCTCTGCGAAAGCCGGTACCGGGATTGATGACTTGCTGAATGCCATCCTGCTGCAGGCGGAAGTTCTTGAACTGACCGCCGTACGTCAGGGCATGGCAAGCGGTGTGGTGATCGAATCCTTCCTGGATAAAGGCCGTGGCCCGGTGGCTACCGTGCTGGTGCGTGAAGGTACGCTGAACAAAGGCGATATCGTGCTGTGTGGTTTCGAATATGGCCGCGTACGAGCAATGCGTGACGAGCTGGGCCGCGAAGTACTGACTGCGGGTCCATCTATCCCGGTTGAGATCCTCGGCATGTCCGGCGTACCGGCGGCGGGTGATGAAGCCACCGTGGTGCGTGACGAGAAGAAAGCGCGTGAAGTGGCGCTGTACCGTCAGGGCAAATTCCGTGAAGTT | GAAGCCATCCAGCATGCGAAAGCGGCGAAAGTGCCGGTTGTGGTGGCGGTAAACAAATGTGATAAGCCAGAAGCCGATCCGGACCGTGTCAAAAACGAACTCACCCAGTACGGCATCATTCCGGAAGAGTGGGGCGGTGAAAACATGTTCGTCAACGTCTCTGCGAAAGCCGGTACCGGGATTGATGACTTGCTGAATGCCATCCTGCTGCAGGCGGAAGTTCTTGAACTGACCGCCGTACGTCAGGGCATGGCAAGCGGTGTGGTGATCGAATCCTTCCTGGATAAAGGCCGTGGCCCGGTGGCTACCGTGCTGGTGCGTGAAGGTACGCTGAACAAAGGCGATATCGTGCTGTGTGGTTTCGAATATGGCCGCGTACGAGCAATGCGTGACGAGCTGGGCCGCGAAGTACTGACTGCGGGTCCATCTATCCCGGTTGAGATCCTCGGCATGTCCGGCGTACCGGCGGCGGGTGATGAAGCCACCGTGGTGCGTGACGAGAAGAAAGCGCGTGAAGTGGCGCTGTACCGTCAGGGCAAATTCCGTGAAGTT |
| ***rpoD* Sequence** | CGATTACTTATCTGCTTGAACAGTATGATCGTGTTGAAGCAGGTGAAGCGCGCCTGTCCGATCTGATCACCGGCTTCGTCGATCCTAACGCTGAAGAAGATATTGCCCCGACCGCTACGCACGTAGGTTCTGAGCTGTCTGCGGAAGAGCGTGACGACGACGAAGATGAAGACGAAGAATCTGACGACGACAGCTCGGATGATGACAACAGCATAGATCCTGAGCTGGCGCGTGAAAAGTTCAACGATTTGCGCATCCAGTACGAAACCACCCGTACGGTGATTAAAGCTAAAAGCCGCAGTCACGCTGATGCCATCGCAGAGATCCAGAATCTGTCCGACGTATTTAAACAGTTCCGCCTGGTGCCGAAGCAGTTCGACTTCCTGGTGAACAGCATGCGTGTCATGATGGATCGCGTTCGTACTCAGGAACGTATCATCATGAAGCTGTGTGTTGAATTGTGCAAAATGCCGAAGAAAAACTTCATCACGCTGTTTACCGGCAATGAGACCAGTGAAACCTGGTTCAAAGCCGCGCTGGCAATGAACAAACCGTGGTCGGAGAAGCTGAACGATGTTTCAGAAGATGTGAACCGCGGCCTGCAGAAGCTGCAGCAGATT | CGATTACTTATCTGCTTGAACAGTATGATCGTGTTGAAGCAGGTGAAGCGCGCCTGTCCGATCTGATCACCGGCTTCGTCGATCCTAACGCTGAAGAAGATATTGCCCCGACCGCTACGCACGTAGGTTCTGAGCTGTCTGCGGAAGAGCGTGACGACGACGAAGATGAAGACGAAGAATCTGACGACGACAGCTCGGATGATGACAACAGCATAGATCCTGAGCTGGCGCGTGAAAAGTTCAACGATTTGCGCATCCAGTACGAAACCACCCGTACGGTGATTAAAGCTAAAAGCCGCAGTCACGCTGATGCCATCGCAGAGATCCAGAATCTGTCCGACGTATTTAAACAGTTCCGCCTGGTGCCGAAGCAGTTCGACTTCCTGGTGAACAGCATGCGTGTCATGATGGATCGCGTTCGTACTCAGGAACGTATCATCATGAAGCTGTGTGTTGAATTGTGCAAAATGCCGAAGAAAAACTTCATCACGCTGTTTACCGGCAATGAGACCAGTGAAACCTGGTTCAAAGCCGCGCTGGCAATGAACAAACCGTGGTCGGAGAAGCTGAACGATGTTTCAGAAGATGTGAACCGCGGCCTGCAGAAGCTGCAGCAGATT | CGATTACTTATCTGCTTGAACAGTATGATCGTGTTGAAGCAGGTGAAGCGCGCCTGTCCGATCTGATCACCGGCTTCGTCGATCCTAACGCTGAAGAAGATATTGCCCCGACCGCTACGCACGTAGGTTCTGAGCTGTCTGCGGAAGAGCGTGACGACGACGAAGATGAAGACGAAGAATCTGACGACGACAGCTCGGATGATGACAACAGCATAGATCCTGAGCTGGCGCGTGAAAAGTTCAACGATTTGCGCATCCAGTACGAAACCACCCGTACGGTGATTAAAGCTAAAAGCCGCAGTCACGCTGATGCCATCGCAGAGATCCAGAATCTGTCCGACGTATTTAAACAGTTCCGCCTGGTGCCGAAGCAGTTCGACTTCCTGGTGAACAGCATGCGTGTCATGATGGATCGCGTTCGTACTCAGGAACGTATCATCATGAAGCTGTGTGTTGAATTGTGCAAAATGCCGAAGAAAAACTTCATCACGCTGTTTACCGGCAATGAGACCAGTGAAACCTGGTTCAAAGCCGCGCTGGCAATGAACAAACCGTGGTCGGAGAAGCTGAACGATGTTTCAGAAGATGTGAACCGCGGCCTGCAGAAGCTGCAGCAGATT | CGATTACTTATCTGCTTGAACAGTATGATCGTGTTGAAGCAGGTGAAGCGCGCCTGTCCGATCTGATCACCGGCTTCGTCGATCCTAACGCTGAAGAAGATATTGCCCCGACCGCTACGCACGTAGGTTCTGAGCTGTCTGCGGAAGAGCGTGACGACGACGAAGATGAAGACGAAGAATCTGACGACGACAGCTCGGATGATGACAACAGCATAGATCCTGAGCTGGCGCGTGAAAAGTTCAACGATTTGCGCATCCAGTACGAAACCACCCGTACGGTGATTAAAGCTAAAAGCCGCAGTCACGCTGATGCCATCGCAGAGATCCAGAATCTGTCCGACGTATTTAAACAGTTCCGCCTGGTGCCGAAGCAGTTCGACTTCCTGGTGAACAGCATGCGTGTCATGATGGATCGCGTTCGTACTCAGGAACGTATCATCATGAAGCTGTGTGTTGAATTGTGCAAAATGCCGAAGAAAAACTTCATCACGCTGTTTACCGGCAATGAGACCAGTGAAACCTGGTTCAAAGCCGCGCTGGCAATGAACAAACCGTGGTCGGAGAAGCTGAACGATGTTTCAGAAGATGTGAACCGCGGCCTGCAGAAGCTGCAGCAGATT | CGATTACTTATCTGCTTGAACAGTATGATCGTGTTGAAGCAGGTGAAGCGCGCCTGTCCGATCTGATCACCGGCTTCGTCGATCCTAACGCTGAAGAAGATATTGCCCCGACCGCTACGCACGTAGGTTCTGAGCTGTCTGCGGAAGAGCGTGACGACGACGAAGATGAAGACGAAGAATCTGACGACGACAGCTCGGATGATGACAACAGCATAGATCCTGAGCTGGCGCGTGAAAAGTTCAACGATTTGCGCATCCAGTACGAAACCACCCGTACGGTGATTAAAGCTAAAAGCCGCAGTCACGCTGATGCCATCGCAGAGATCCAGAATCTGTCCGACGTATTTAAACAGTTCCGCCTGGTGCCGAAGCAGTTCGACTTCCTGGTGAACAGCATGCGTGTCATGATGGATCGCGTTCGTACTCAGGAACGTATCATCATGAAGCTGTGTGTTGAATTGTGCAAAATGCCGAAGAAAAACTTCATCACGCTGTTTACCGGCAATGAGACCAGTGAAACCTGGTTCAAAGCCGCGCTGGCAATGAACAAACCGTGGTCGGAGAAGCTGAACGATGTTTCAGAAGATGTGAACCGCGGCCTGCAGAAGCTGCAGCAGATT | CGATTACTTATCTGCTTGAACAGTATGATCGTGTTGAAGCAGGTGAAGCGCGCCTGTCCGATCTGATCACCGGCTTCGTCGATCCTAACGCTGAAGAAGATATTGCCCCGACCGCTACGCACGTAGGTTCTGAGCTGTCTGCGGAAGAGCGTGACGACGACGAAGATGAAGACGAAGAATCTGACGACGACAGCTCGGATGATGACAACAGCATAGATCCTGAGCTGGCGCGTGAAAAGTTCAACGATTTGCGCATCCAGTACGAAACCACCCGTACGGTGATTAAAGCTAAAAGCCGCAGTCACGCTGATGCCATCGCAGAGATCCAGAATCTGTCCGACGTATTTAAACAGTTCCGCCTGGTGCCGAAGCAGTTCGACTTCCTGGTGAACAGCATGCGTGTCATGATGGATCGCGTTCGTACTCAGGAACGTATCATCATGAAGCTGTGTGTTGAATTGTGCAAAATGCCGAAGAAAAACTTCATCACGCTGTTTACCGGCAATGAGACCAGTGAAACCTGGTTCAAAGCCGCGCTGGCAATGAACAAACCGTGGTCGGAGAAGCTGAACGATGTTTCAGAAGATGTGAACCGCGGCCTGCAGAAGCTGCAGCAGATT | CGATTACTTATCTGCTTGAACAGTATGATCGTGTTGAAGCAGGTGAAGCGCGCCTGTCCGATCTGATCACCGGCTTCGTCGATCCTAACGCTGAAGAAGATATTGCCCCGACCGCTACGCACGTAGGTTCTGAGCTGTCTGCGGAAGAGCGTGACGACGACGAAGATGAAGACGAAGAATCTGACGACGACAGCTCGGATGATGACAACAGCATAGATCCTGAGCTGGCGCGTGAAAAGTTCAACGATTTGCGCATCCAGTACGAAACCACCCGTACGGTGATTAAAGCTAAAAGCCGCAGTCACGCTGATGCCATCGCAGAGATCCAGAATCTGTCCGACGTATTTAAACAGTTCCGCCTGGTGCCGAAGCAGTTCGACTTCCTGGTGAACAGCATGCGTGTCATGATGGATCGCGTTCGTACTCAGGAACGTATCATCATGAAGCTGTGTGTTGAATTGTGCAAAATGCCGAAGAAAAACTTCATCACGCTGTTTACCGGCAATGAGACCAGTGAAACCTGGTTCAAAGCCGCGCTGGCAATGAACAAACCGTGGTCGGAGAAGCTGAACGATGTTTCAGAAGATGTGAACCGCGGCCTGCAGAAGCTGCAGCAGATT |
