# Supplementary material for: Variability within a clonal population of Erwinia amylovora disclosed by phenotypic analysis
Source: PeerJ. 2022 Jul 21;10:e13695. doi: 10.7717/peerj.13695 (PMC9308965; doi:10.7717/peerj.13695)
Supplement: Supplemental Information 5 — Substrates: Black - Carbon source utilization assay/Red - Chemical susceptibly assay. Colour indicates positive (violet), negative (white) or borderline (light violet) [file peerj-10-13695-s005.docx]

**Table S4.** Biochemical pattern obtained for the seven Portuguese Erwinia amylovora strains, type strain LMG 2024 and reference strain CFBP 1430 by BIOLOG GEN III. Substrates: Black - Carbon source utilization assay / Red - Chemical susceptibly assay. Colour indicates positive (violet), negative (white) or borderline (light violet).

|  |  |  |  |  |  |  |  |  |  |
| --- | --- | --- | --- | --- | --- | --- | --- | --- | --- |
| **Substrate^a^** | **LMG 2024** | **Ea 230** | **Ea 320** | **Ea 390** | **Ea 490** | **Ea 630** | **Ea 680** | **Ea 820** | **CFBP 1430** |
| **D-Raffinose** |  |  |  |  |  |  |  |  |  |
| **α-D-Glucose** |  |  |  |  |  |  |  |  |  |
| **D-Sorbitol** |  |  |  |  |  |  |  |  |  |
| **Gelatin** |  |  |  |  |  |  |  |  |  |
| **Pectin** |  |  |  |  |  |  |  |  |  |
| **p-Hydroxy-Phenylacetic Acid** |  |  |  |  |  |  |  |  |  |
| **Tween 40** |  |  |  |  |  |  |  |  |  |
| **Dextrin** |  |  |  |  |  |  |  |  |  |
| **α-D-Lactose** |  |  |  |  |  |  |  |  |  |
| **D-Mannose** |  |  |  |  |  |  |  |  |  |
| **D-Mannitol** |  |  |  |  |  |  |  |  |  |
| **Glycyl-L-Proline** |  |  |  |  |  |  |  |  |  |
| **D-Galacturonic Acid** |  |  |  |  |  |  |  |  |  |
| **Methyl Pyruvate** |  |  |  |  |  |  |  |  |  |
| **γ-Amino-Butryric Acid** |  |  |  |  |  |  |  |  |  |
| **D-Maltose** |  |  |  |  |  |  |  |  |  |
| **D-Melibiose** |  |  |  |  |  |  |  |  |  |
| **D-Fructose** |  |  |  |  |  |  |  |  |  |
| **D-Arabitol** |  |  |  |  |  |  |  |  |  |
| **L-Alanine** |  |  |  |  |  |  |  |  |  |
| **L-Galactonic Acid Lactone** |  |  |  |  |  |  |  |  |  |
| **D-Lactic Acid Methyl Ester** |  |  |  |  |  |  |  |  |  |
| **α-Hydroxy-Butyric Acid** |  |  |  |  |  |  |  |  |  |
| **D-Trehalose** |  |  |  |  |  |  |  |  |  |
| **β-Methyl-D-Glucoside** |  |  |  |  |  |  |  |  |  |
| **D-Galactose** |  |  |  |  |  |  |  |  |  |
| **myo-Inositol** |  |  |  |  |  |  |  |  |  |
| **L-Arginine** |  |  |  |  |  |  |  |  |  |
| **D-Gluconic Acid** |  |  |  |  |  |  |  |  |  |
| **L-Lactic Acid** |  |  |  |  |  |  |  |  |  |
| **β-Hydroxy-D,L-Butyric Acid** |  |  |  |  |  |  |  |  |  |
| **D-Cellobiose** |  |  |  |  |  |  |  |  |  |
| **D-Salicin** |  |  |  |  |  |  |  |  |  |
| **3-Methyl Glucose** |  |  |  |  |  |  |  |  |  |
| **Glycerol** |  |  |  |  |  |  |  |  |  |
| **L-Aspartic Acid** |  |  |  |  |  |  |  |  |  |
| **D-Glucuronic Acid** |  |  |  |  |  |  |  |  |  |
| **Citric Acid** |  |  |  |  |  |  |  |  |  |
| **α-Keto-Butyric Acid** |  |  |  |  |  |  |  |  |  |
| **Gentiobiose** |  |  |  |  |  |  |  |  |  |
| **N-Acetyl-D-Glucosamine** |  |  |  |  |  |  |  |  |  |
| **D-Fucose** |  |  |  |  |  |  |  |  |  |
| **D-Glucose-6-PO4** |  |  |  |  |  |  |  |  |  |
| **L-Glutamic Acid** |  |  |  |  |  |  |  |  |  |
| **Glucuronamide** |  |  |  |  |  |  |  |  |  |
| **α-Keto-Glutaric Acid** |  |  |  |  |  |  |  |  |  |
| **Acetoacetic Acid** |  |  |  |  |  |  |  |  |  |
| **Sucrose** |  |  |  |  |  |  |  |  |  |
| **N-Acetyl-β-D-Mannosamine** |  |  |  |  |  |  |  |  |  |
| **L-Fucose** |  |  |  |  |  |  |  |  |  |
| **D-Fructose-6-PO4** |  |  |  |  |  |  |  |  |  |
| **L-Histidine** |  |  |  |  |  |  |  |  |  |
| **Mucic Acid** |  |  |  |  |  |  |  |  |  |
| **D-Malic Acid** |  |  |  |  |  |  |  |  |  |
| **Propionic Acid** |  |  |  |  |  |  |  |  |  |
| **D-Turanose** |  |  |  |  |  |  |  |  |  |
| **N-Acetyl-D-Galactosamine** |  |  |  |  |  |  |  |  |  |
| **L-Rhamnose** |  |  |  |  |  |  |  |  |  |
| **D-Aspartic Acid** |  |  |  |  |  |  |  |  |  |
| **L-Pyroglutamic Acid** |  |  |  |  |  |  |  |  |  |
| **Quinic Acid** |  |  |  |  |  |  |  |  |  |
| **L-Malic Acid** |  |  |  |  |  |  |  |  |  |
| **Acetic Acid** |  |  |  |  |  |  |  |  |  |
| **Stachyose** |  |  |  |  |  |  |  |  |  |
| **N-Acetyl Neuraminic Acid** |  |  |  |  |  |  |  |  |  |
| **Inosine** |  |  |  |  |  |  |  |  |  |
| **D-Serine** |  |  |  |  |  |  |  |  |  |
| **L-Serine** |  |  |  |  |  |  |  |  |  |
| **D-Saccharic Acid** |  |  |  |  |  |  |  |  |  |
| **Bromo-Succinic Acid** |  |  |  |  |  |  |  |  |  |
| **Formic Acid** |  |  |  |  |  |  |  |  |  |
| **1% NaCl** |  |  |  |  |  |  |  |  |  |
| **1% Sodium Lactate** |  |  |  |  |  |  |  |  |  |
| **Troleandomycin** |  |  |  |  |  |  |  |  |  |
| **Lincomycin** |  |  |  |  |  |  |  |  |  |
| **Vancomycin** |  |  |  |  |  |  |  |  |  |
| **Nalidixic Acid** |  |  |  |  |  |  |  |  |  |
| **Aztreonam** |  |  |  |  |  |  |  |  |  |
| **pH 6** |  |  |  |  |  |  |  |  |  |
| **4% NaCl** |  |  |  |  |  |  |  |  |  |
| **Fusidic Acid** |  |  |  |  |  |  |  |  |  |
| **Rifamycin SV** |  |  |  |  |  |  |  |  |  |
| **Guanidine HCl** |  |  |  |  |  |  |  |  |  |
| **Tetrazolium Violet** |  |  |  |  |  |  |  |  |  |
| **Lithium Chloride** |  |  |  |  |  |  |  |  |  |
| **Sodium Butyrate** |  |  |  |  |  |  |  |  |  |
| **pH 5** |  |  |  |  |  |  |  |  |  |
| **8% NaCl** |  |  |  |  |  |  |  |  |  |
| **D-Serine** |  |  |  |  |  |  |  |  |  |
| **Minocycline** |  |  |  |  |  |  |  |  |  |
| **Niaproof 4** |  |  |  |  |  |  |  |  |  |
| **Tetrazolium Blue** |  |  |  |  |  |  |  |  |  |
| **Potassium Tellurite** |  |  |  |  |  |  |  |  |  |
| **Sodium Bromate** |  |  |  |  |  |  |  |  |  |
